# Anti-prostate cancer mechanism of black ginseng during the "nine steaming and nine sun-drying" process based on HPLC analysis combined with vector space network pharmacology

**DOI:** 10.1007/s12672-024-00862-z

**Published:** 2024-01-18

**Authors:** Youran Zhang, Ye Huang, Deqiang Dou

**Affiliations:** Liaoning University of Traditional Chinese Medicine, 77 Life One Road, DD Port, Dalian, 116600 China

**Keywords:** Black ginseng, Nine steam and nine sun-drying, Multivariate statistical analysis, Network pharmacology

## Abstract

HPLC analysis determined six small-molecule organic acids, maltol, 5-hydroxymethylfurfural (5-HMF), 17 ginsenosides, four oligosaccharides, and 20 amino acids in black ginseng samples with different processing times. Based on the content determination results, the differential ingredients in the processing of black ginseng were screened by multivariate statistical analysis. Network pharmacological methods obtained the core targets and pathways of the above ingredients against prostate cancer. Finally, the entropy weight method was used to assign values to the above ingredients, targets, and pathways, and the vector space network pharmacology method was established to study the anti-prostate cancer mechanism of black ginseng in the process of "nine steaming and nine sun-drying". Based on principal component analysis (PCA) and orthogonal partial least squares discriminant analysis (OPLS-DA), fructose, glucose, dencichin, glutamate, ginsenoside 20 (S)-Rg3, 20 (R)-Rg3, 20 (S)-Rh2, Rg1, Re, and Rc were the main differential ingredients in various steaming and sun-drying cycle periods of black ginseng. The results of vector space network pharmacology showed that the main reason for the change in the anti-prostate cancer pathway of black ginseng with the number of steaming and sun-drying was the different regulatory ability of black ginseng on the PI3K-Akt signaling pathway and chemical carcinogenesis-receptor activation pathway. It gave researchers a fresh perspective for exploring the anti-prostate cancer active components of black ginseng and the change in the mechanism of the effect of traditional Chinese medicine in processing.

## Introduction

Ginseng is the dried root and rhizome of ginseng (*Panax ginseng* C. A. Mey.) in the family Araliaceae, which has the efficacy of tonifying vital energy and generating fluids to quench thirst. Modern pharmacological investigations have demonstrated that ginseng has immune-boosting, blood vessel protection, anti-cancer, and cardiovascular properties [1, 2]. Black ginseng is made by processing ginseng under "nine steaming and nine sun-drying" to produce rare saponins with potent anticancer activity [3]. Recent studies have shown that black ginseng has anticancer [4], antioxidant [5], and immunomodulatory effects [6]. Black ginseng has a better impact against prostate cancer, according to previous research on the variations in the composition of ginseng, red ginseng, and black ginseng[7, 8]. However, the exact foundation for this action is still unknown.

"Nine steaming and nine sun-drying" refers to an ancient method of Traditional Chinese Medicine (TCM) to enhance efficacy, improve the taste, and lessen toxicity by repeatedly steaming and sun-drying[9, 10]. A novel method for determining the active ingredients in herbs can be developed based on the correlation analysis of chemical composition and changes in the efficacy of TCM throughout processing. Network pharmacology is the analytical approach of integrating medications, targets, and bioinformatic networks, which combines well with the multi-component and multi-target synergistic effects of TCM [11, 12]. However, because the network pharmacology analytical approach depends on already-existing databases and does not account for the content of chemical ingredients in herbs, the accuracy of the analysis results is inaccurate. The entropy weighting method (EWM) is an objective weighting method that calculates weights based on the information entropy (IE) of the attributes [13], thus avoiding the influence of subjective preferences on the assessment results. It has been widely used in medicine and pharmacy [14–16]. In order to properly characterize the link between chemical ingredients in herbs and their efficacy and to clarify their mechanism of action, it is required to establish a multidimensional and accurate analytical approach by combining different analytical methods.

In summary, in order to investigate the mechanism of the anti-prostate cancer effects of black ginseng during the "nine steaming and nine sun-drying" process, this study first quantified six key chemical ingredients, including organic acids, maltol, 5-HMF, ginsenosides, oligosaccharides, and amino acids. PCA and OPLS-DA were used to screen for the principal differential ingredients in anti-prostate cancer based on the contents of the ingredients in the samples in various steaming and sun-drying cycle periods. Finally, a vector space network pharmacological analysis method was established with network pharmacology and the entropy weight method to determine the anti-prostate cancer mechanism of black ginseng in the process of "nine steam and nine sun-drying".

## Materials and methods

### Materials and reagents

Samples of White ginseng and "nine steaming and nine sun-drying" black ginseng were purchased from Liaoning Zhongshutang Black Ginseng Co. (TieLing, China). The standard white and black ginseng extracts were prepared in the laboratory [17]. Maltol was acquired from J&K Scientific (Beijing, China). 5-hydroxymethylfurfural (5-HMF), D-malic acid, L-malic acid, acetic acid, malonic acid, and succinic acid were procured from Fluka (Seelze, Germany). Citric acid was procured from Sigma (St. Louis, Missouri, USA). Eighteen amino acids, derivatization reagents (2,4-dinitrofluorobenzene), and solid components A and B were acquired from Elite Company (Dalian, China). γ-aminobutyric acid (GABA), dencichin, and dextrose were procured from Dr. Ehrenstorfer (Germany). Fructose, maltose, and sucrose were procured from Wako (Japan). Glacial acetic acid, N, N-dimethylformamide, and phosphoric acid were procured from Komeo (Tianjin, China). Purified water was procured from Watson's (Hong Kong, China). HPLC-grade methanol and acetonitrile were obtained from Oceanpak and Tedia (Fairfield, OH, USA).

### Preparation of aqueous extract

Three samples were taken from each batch of black ginseng S0D0 ~ S9D9 (S for steaming; D for sun-drying; the number represents the number of cycles of steaming and sun-drying). Dried, mixed, and crushed, passed through sieve No. 4, placed in a round-bottomed flask, added 25 times the amount of water, and soaked overnight. Extracted twice for 2 h each time at reflux, filtered, combined filtrate, and concentrated. It was concentrated and freeze-dried for 72 h to create the black ginseng aqueous extracts, and they were then kept in a dry environment at 4 °C. The lyophilized powder of the extract was dissolved in purified water and filtered through a 0.22 µm microporous membrane before HPLC analysis.

### Determination of ingredients in black ginseng

#### Determination of organic acids, maltol, 5-HMF, and ginsenosides

The content of organic acids, maltol, 5-HMF, and ginsenosides in black ginseng was determined by reference to organic acids [18], maltol [19], 5-HMF [20], and the ginsenoside determination method [21]. An exact weight of 1 g of the sample powder was taken, dissolved to 100 mg/mL in purified water while vigorously shaking the mixture, and filtered through a 0.22 m microporous membrane to get the test solution. HPLC analysis was performed using an Agilent 1260 HPLC equipped with a DAD detector. An Agilent ZORBAX SB-Aq (250 mm × 4.6 mm, 5 μm) column was used and maintained at 35 °C. The flow rate was performed as follows: 0 ~ 6 min, 0.5 ~ 1.2 mL/min; 6 ~ 60 min, 1.2 mL/min, with an injection volume of 10 μL. Using acetonitrile (A) and 0.1% phosphoric acid water (B) as mobile phases, gradient elution was optimized as follows: 0 ~ 6 min, 100% (B); 6 ~ 11 min, 100% ~ 95% (B); 11 ~ 16 min, 95% ~ 79% (B); 16 ~ 30 min, 79% (B); 30 ~ 34 min, 79% ~ 70% (B); 34 ~ 39 min, 70% (B); 39 ~ 40 min, 70% ~ 66% (B); 40 ~ 45 min, 66%(B); 45 ~ 48 min, 66% ~ 53% (B); 48 ~ 58 min, 53% ~ 5% (B); 58 ~ 60 min, 5%(B). The detection wavelength was set at: 0 min, 210 nm; 10 min, 284 nm; 27 min, 203 nm; 32 min, 360 nm; 37 min, 203 nm.

#### Determination of oligosaccharides

Referring to the oligosaccharide determination method [17] to determine the oligosaccharide content of black ginseng. An exact weight of 0.5 g of the sample powder was taken, dissolved to 50 mg/mL in purified water while vigorously shaking the mixture, and filtered through a 0.22 m microporous membrane to get the test solution. An Agilent ZORBAX NH_2_ (4.6 × 150 mm, 5 μm) column was used and maintained at 30 °C. Using acetonitrile (A) and water (B) as mobile phases, gradient elution was optimized as follows: 0 ~ 5 min, 12% ~ 17% (B); 5 ~ 10 min, 17% (B); 10 ~ 20 min, 17% ~ 28% (B); 20 ~ 30 min, 28% ~ 33% (B). The flow rate was 1 mL/min with an injection volume of 10 μL. The detector was ELSD, with a drift tube temperature of 40 °C; the carrier gas was nitrogen; and the evaporative light detector gain was 5 °C.

#### Determination of 18 protein amino acids

Referring to the amino acid analysis system (Dalian Elite) [22], the samples to be tested were prepared by pre-column derivatization, and an ODS (4.6 mm × 250 mm, 5 μm, Dalian Elite) column with a DAD detector was selected for the determination of the samples according to the amino acid analysis system.

#### Determination of GABA

The samples to be tested were prepared by pre-column derivatization using the reference amino acid analysis system (Dalian Elite) [23]. An Agilent ZORBAX SB-Aq (250 mm × 4.6 mm, 5 μm) column was used and maintained at 30 °C. The detector was DAD, and the detection wavelength was 360 nm. Using acetonitrile (A) and 0.1% phosphoric acid water (B) as mobile phases, gradient elution was optimized as follows: 0 ~ 7 min, 85% ~ 60% (B); 7 ~ 8.5 min, 60% (B); 8.5 ~ 11 min, 60% ~ 50% (B); 11 ~ 15 min, 50% ~ 40% (B); 15 ~ 20 min, 40% ~ 30% (B); 20 ~ 23 min, 30% ~ 20% (B); 23 ~ 24 min, 20% ~ 0% (B); 24 ~ 28 min, 0% (B). The flow rate was 1 mL/min with an injection volume of 10 μL.

#### Determination of Dencichin

Refer to the determination of dencichin [24]. An exact weight of 1 g of the sample powder was taken, dissolved to 100 mg/mL in purified water while vigorously shaking the mixture, and filtered through a 0.22 m microporous membrane to get the test solution. ODS (4.6 mm × 250 mm, 5 μm, Dalian Elite) column was used and maintained at 30 °C. The detector was DAD, and the detection wavelength was 213 nm. The flow rate was 0.6 mL/min with an injection volume of 5 μL. The mobile phase consisted of acetonitrile (A) and 0.1% phosphoric acid water (B) with the following optimized gradient elution: 0 ~ 5 min, 85% ~ 50% (B); 5 ~ 10 min, 50% ~ 25% (B); 10 ~ 15 min, 25% ~ 5% (B);15 ~ 17 min, 5% (B).

### Multivariate statistical analysis

In SIMCA14.1 software, the PCA and OPLS-DA analyses were performed on the contents of the ingredients of black ginseng during the "nine steaming and nine sun-drying" process. PCA is a technique for consolidating multiple indicators and resolving the covariance issue through mathematical dimensionality reduction. As these composite indicators are independent of one another and can retain most of the information of the original indicators, this effectively prevents interference from repetitive information [25]. Based on the PCA and PLS-DA analysis approach, OPLS-DA analysis includes orthogonal transformation correction, which may divide the X matrix into two types of information relevant to Y and irrelevant information and filter the difference variables by eliminating the irrelevant differences [26].

### Vector spatial network pharmacological analysis

#### Network pharmacological analysis

Combining the PCA and OPLS-DA results, ten differential ingredients with top VIP values were screened. The ingredients were uploaded to the SwissTargetPrediction database (http://swisstargetprediction.ch), and the targets were predicted by the reverse pharmacophore matching method. The molecules with a target probability value (Probability) ≥ 0 were taken as the effective targets of the ingredient and corrected by the UniProt database (https://www.uniprot.org). The GeneCards (https://www.genecards.org) and OMIM (https://www.omim.org) databases were mined for related genes associated with prostate cancer. The effective targets of all the differential components contained in S0D0 ~ S9D9 were combined, respectively, and a Venn diagram was created to identify the shared herb and disease targets. The above-intersected targets were uploaded to the STRING database (https://string-db.org) to obtain the PPI network, and the Degree value of each target was obtained using Cytoscape software Analyze Network and cytoNCA analysis. The Metascape database (https://metascape.org) was used to analyze the KEGG pathway of the targets.

#### Entropy weighting method assignment

The entropy weighting method was used to assign values to the differential ingredients, targets, and KEGG pathways using Eqs. 1  ~ 8 to determine the specific pathways of black ginseng against prostate cancer. The results are first normalized to the data. Assume that there are *n* samples, each with *k* evaluation indicators, thus forming a sequence of raw data units {*Xij*} (i = 1, 2, 3…n; j = 1, 2, 3...k; i = 10, j = 10 in this study). Equation (1) was used to standardize the raw data. *Yij* is the standardized data, *Xij* is the jth index value of the ith sample, and *{Xj}* is the data series of the jth index [27, 28].

1 $${Y}_{ij}=\frac{{X}_{ij}-min\{{X}_{j}\}}{max\{{X}_{j}\}-min\{{X}_{j}\}}$$

In general, an attribute's smaller IE indicates a greater degree of diversity or separateness of the attribute, a greater role it plays in the assessment, and a greater weight. Calculate the IE of each index using Eqs. (2) and (3), according to the definition of information entropy in information theory. *Ej* is the IE of the jth index of the sample; if *P*_*ij*_ = 0, then *lnP*_*ij*_ = 0 is defined.

2 $${P}_{ij}=\frac{{Y}_{ij}}{\sum_{i=1}^{n}{Y}_{ij}}\cdot$$

3 $${E}_{j}=\frac{1}{\mathit{ln}n}\sum_{i=1}^{n}{P}_{ij}\mathit{ln}({P}_{ij})$$

Finally, the weight of each index (*Wj*) was calculated according to Eq. (4).

4 $${W}_{j}=\frac{1-{E}_{j}}{\sum_{i=1}^{n}{(1-E}_{j})}.$$

The *Wj* of the ten differential ingredients in S0D0 ~ S9D9 were weighted with their contents in the samples in various steaming and sun-drying cycle periods. The ingredient's final assigned value (*ρ*) in SnDn was obtained according to Eq. (5).

5 $$\rho = Wj \times samples' content$$

By weighting each target by the assignment (*ρ*) of each ingredient and the Degree value of the target in the PPI network, Eq. (6) was utilized to generate the final assignment (*σ*) of each target in SnDn.

6 $$\sigma =\sum_{i=1}^{n}\rho \times Degree$$

The score (*ω*) of each pathway was then thoroughly derived using Eqs. (7) and (8) based on the assignment results of each target and the enrichment of the major anti-prostate cancer pathways. GeneInTerm is the number of targets in the pathway in which the drug treats the disease; count is the total number of targets in which the drug treats the disease.

7 $$GeneRatio=\frac{GeneInTerm}{count}\cdot$$

8 $$\omega =\sum_{i=1}^{n}\sigma \times GeneRatio$$

### Data processing

Liquid phase data were collected using Agilent ChemStation Rev. B.04.03; content determination results were analyzed using SPSS version 26 and were statistically significant at P < 0.05; PCA and OPLS-DA analysis were performed using SIMCA 14.1 software.

## Results and discussion

### Determination of chemical composition

In the detection range, the peak area of each ingredient showed a linear relationship with the concentration (r > 0.9991); the repeatability RSD (n = 6) was < 2.67%; the precision RSD (n = 6) was < 2.22%; the stability was good within 24 h, and the stability RSD (n = 6) was < 2.56%; the recoveries of spiked samples were in the range of 95.3% ~ 103.2%, and the RSD (n = 6) was less than 7.27%. The results showed that the established quantitative method is accurate, reliable, and meets the relevant requirements so that it can be used for the determination of the ingredients of black ginseng [17–24].

**Fig. 1:**
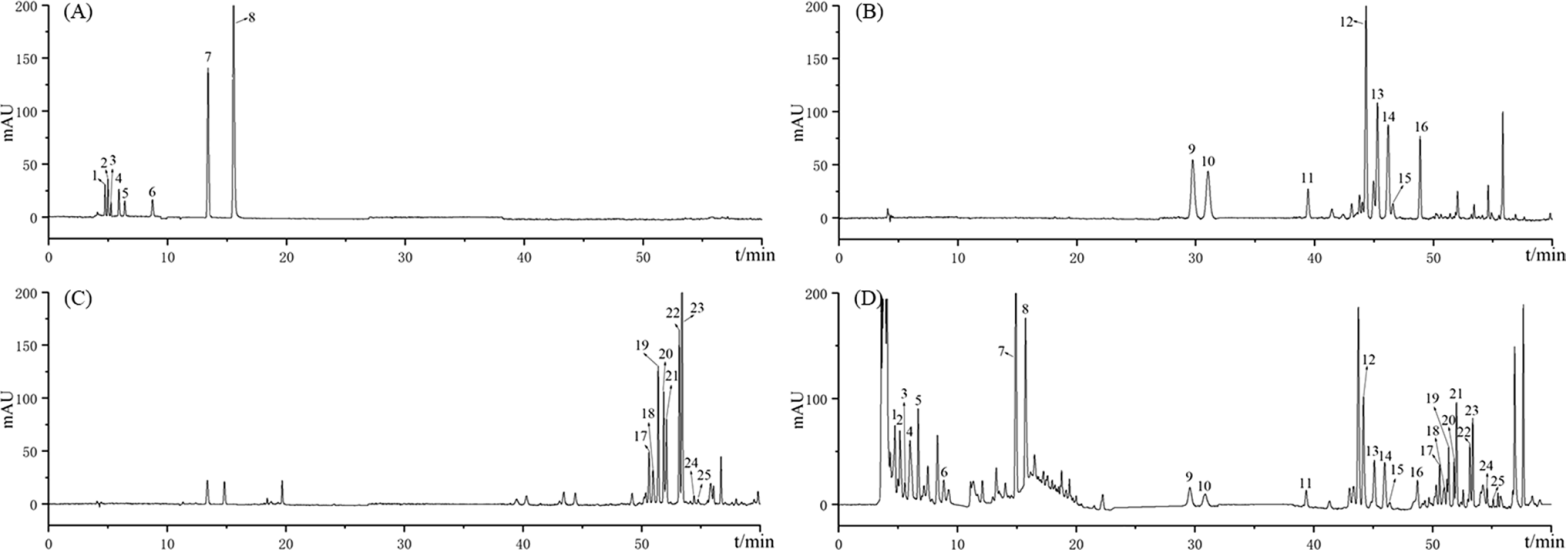
HPLC chromatogram of organic acid, 5-HMF and maltol mixed reference substances (**A**), Standard extract of white ginseng (**B**), Standard extract of black ginseng (**C**), organic acid, 5-HMF, maltol and ginsenosides in S3D3 sample (**D**) 1.D-malic acid; 2.Malonic acid; 3.Acetic acid; 4.Citric acid; 5.Succinic acid; 6.L-Malic acid; 7.5-HMF; 8.Maltol; 9~25.Ginsenoside Rg1, Re, Rf, Rb1, Rc, Rb2, Rb3, Rd, F4, Rk3, Rh4, 20 (S)-Rg3, 20 (R)-Rg3, Rk1, Rg5, 20 (S)-Rh2, 20 (R)-Rh2

**Fig. 2:**
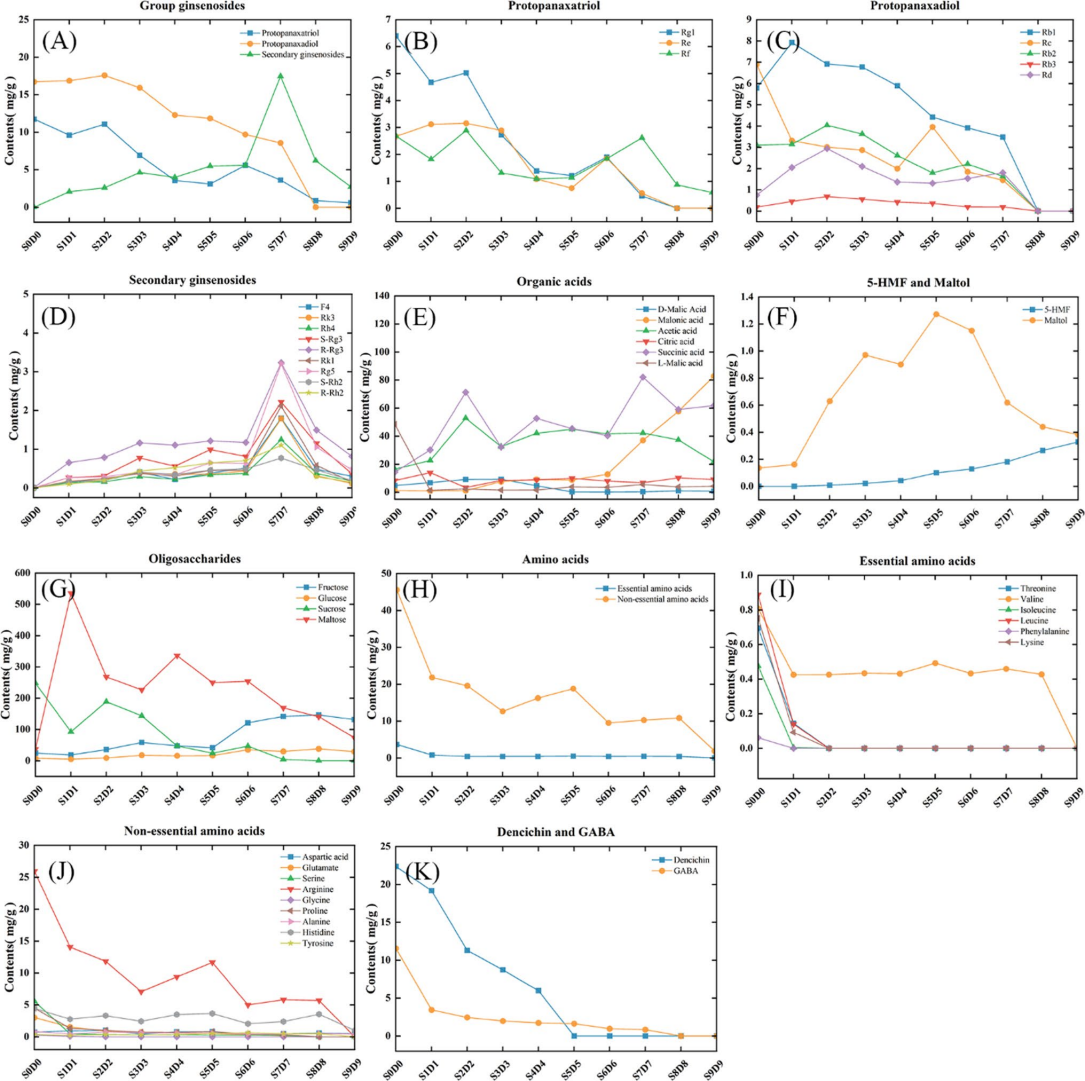
Effect of different cycle times of steaming and sun-drying on ingredients of black ginseng: group ginsenosides (**A**), protopanaxatriol (**B**), protopanaxadiol (**C**), secondary ginsenosides (**D**), organic acids (**E**), 5-HMF and maltol (**F**), oligosaccharides (**G**), amino acids (**H**), essential amino acids (**I**), non-essential amino acids (**J**), dencichin and GABA (**K**)

HPLC results are shown in Fig. 1. P1 ~ P6 were determined to be D-malic acid, malonic acid, acetic acid, citric acid, succinic acid, and L-malic acid, respectively. P7 was 5-HMF. P8 was maltol. P9 ~ P16 were identified as ginsenosides Rg1, Re, Rf, Rb1, Rc, Rb2, Rb3, and Rd, respectively, and were primary ginsenosides. P17 ~ P25 were identified as ginsenosides F4, Rk3, Rh4, 20 (S)-Rg3, 20 (R)-Rg3, Rk1, Rg5, 20 (S)-Rh2, and 20 (R)-Rh2, respectively, and were secondary ginsenosides. The content determination results are shown in Fig. 2. The raw ginseng S0D0 contained only primary ginsenoside. 5-HMF was not detected; a small amount of maltol was detected, and the organic acid and L-malic acid were high at 48.49 mg/g (Fig. 2A–F). S1D1 began to produce secondary ginsenoside, and the content of l-malic acid was drastically reduced to 1.40 mg/g. S2D2 had the highest overall ginsenoside content of 31.23 mg/g, with a higher acetic acid content and a detectable 5-HMF of 0.008 mg/g. Accordingly, S2D2 exhibited a significant chemical difference from S0D0. The content of primary ginsenosides in the samples from S3D3 to S6D6 was gradually decreasing, and the content of secondary ginsenosides, 5-HMF, and malonate was gradually increasing. There was a significant increase in the content of secondary ginsenosides, 5-HMF, and malonate in the S7D7 compared to the S6D6, and some primary ginsenosides could still be detected. The content of secondary ginsenosides in the S7D7 was the highest (17.41 mg/g). The primary ginsenosides were almost completely converted in the samples of S8D8 and S9D9, and the content of secondary ginsenosides was reduced significantly compared to that of S7D7. The content of overall ginsenosides was the lowest in S9D9 (3.29 mg/g), possibly caused by excessive processing. 5-HMF was also found to be the most important in S8D8 and S9D9 and gradually increased with the increase in the number of processing, with the highest content of 5-HMF in S9D9 (0.0291 mg/g).

**Fig. 3:**
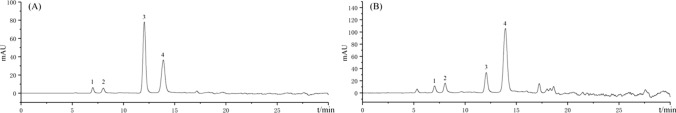
HPLC chromatogram of oligosaccharides reference substances (**A**), oligosaccharides in S3D3 sample (**B**) 1. Fructose; 2. Glucose; 3. Sucrose; 4. Maltose

According to Fig. 3, P1 ~ P4 were determined to be fructose, glucose, sucrose, and maltose, respectively. Fructose and glucose gradually increased with the steaming and sun-drying cycles (Fig. 2G). Sucrose content was highest in S0D0 (247.82 mg/g). Maltose content was highest in S1D1 (535.53 mg/g), and sucrose content was reduced drastically compared to S0D0 [28]. Afterward, the content of both maltose and sucrose gradually decreased.

**Fig. 4:**
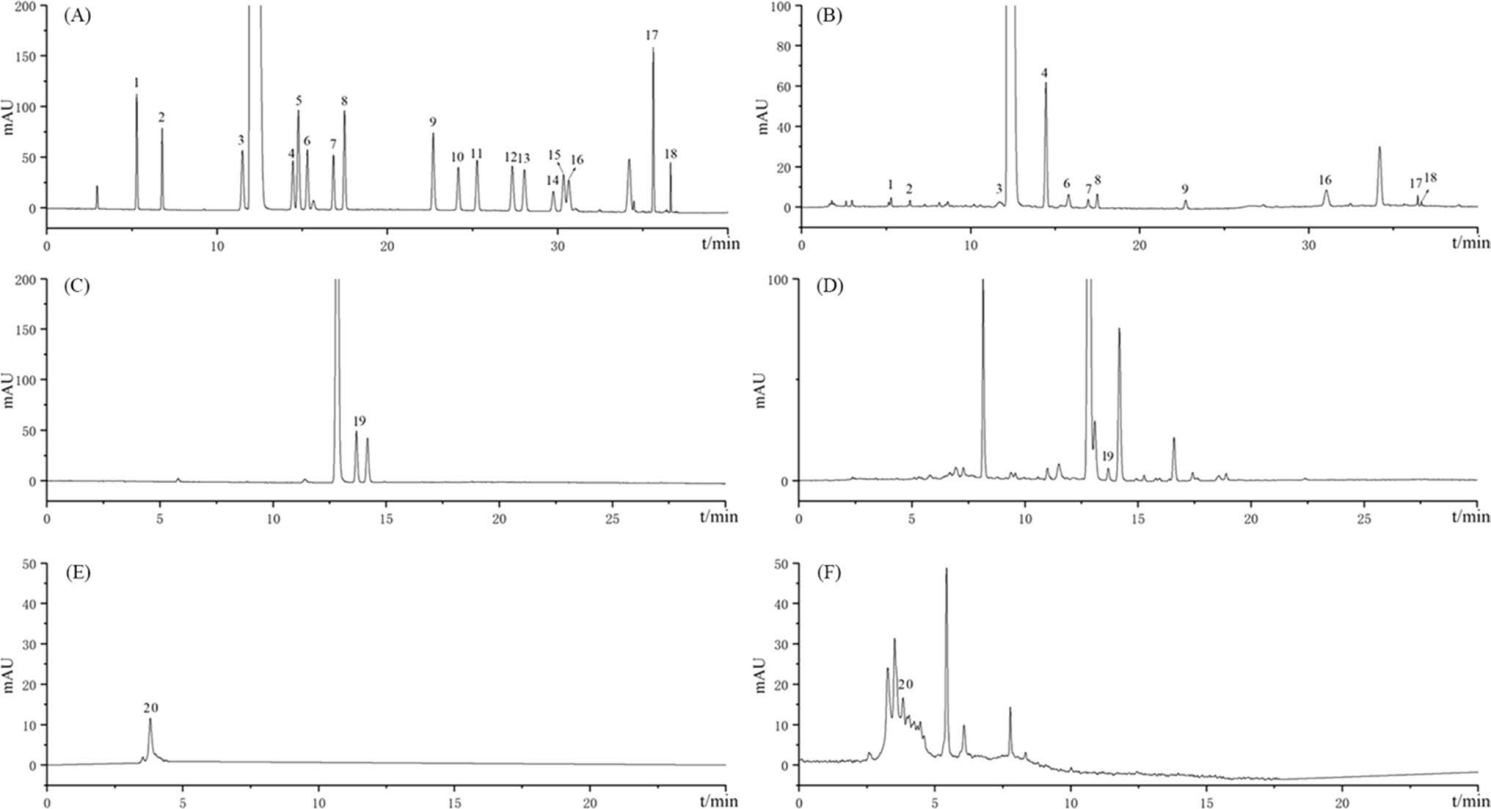
HPLC chromatogram of amino acids reference substances (**A**), amino acids in S3D3 sample (**B**), GABA reference substances (**C**), GABA in S3D3 sample (**D**), Dencichin reference substances (**E**), Dencichin in S3D3 sample (**F**) 1. Aspartic acid; 2. Glutamate; 3. Serine; 4. Arginine; 5. Glycine; 6. Threonine; 7. Proline; 8. Alanine; 9. Valine; 10. Methionine; 11. Cysteine; 12. Isoleucine; 13. Leucine; 14. Tryptophan; 15. Phenylalanine; 16. Histidine; 17. Lysine; 18. Tyrosine; 19. GABA; 20. Dencichin

As shown in Fig. 4, P1 ~ P18 were determined to be 18 protein amino acids, respectively. P19 and P20 were determined to be GABA and dencichin. The total amount of amino acids in the samples showed a gradual decrease with the number of cycles of steaming and sun-drying (Fig. 2H–J). The total amount of essential amino acids tends to stabilize after S2D2. In S9D9, except for aspartic acid, alanine, and histidine, they were no longer detectable. Cysteine, methionine, and tryptophan were not detected in this experiment. The contents of both dencichin and GABA showed a decreasing trend with the increase in the number of cycles of steaming and sun-drying; the content of dencichin decreased to 0 in S5D5, and the content of GABA decreased to 0 in S8D8(Fig. 2K).

Based on the results of HPLC, we found that during the processing, C3 and C20 sugars in ginsenosides Ra, Rb1, Rb2, Rb3, Rc, and Rd were hydrolyzed to generate Rg3 and Rh2; Rg3 generates Rk1 and Rg5 through dehydration reactions; and Rg2 was hydrolyzed and dehydrated to produce Rg6, F4, Rk3, and Rh4 [29]. Consequentially, this caused a steady decrease in the amount of primary ginsenosides in black ginseng, an increase in the amount of secondary ginsenosides, and the primary ginsenosides progressively converged into secondary ginsenosides. As the number of steaming and sun-drying cycle periods increased, sucrose and maltose were continually hydrolyzed into one glucose molecule, one fructose molecule, and two glucose molecules, respectively. Therefore, their content gradually declined while fructose and glucose content steadily grew. The Maillard reaction, a non-enzymatic thermal reaction in the processing of TCM [30], alters the flavor and color of ginseng. One of the byproducts of the Maillard process is the short-molecule aldehyde compound 5-HMF, which possesses antioxidant and neuroprotective properties [31]. Polyphenols are the secondary metabolites of ginseng, and maltol has a high concentration and antioxidant activity [32]. The amounts of 5-HMF and maltol gradually rose as processing cycles increased. Eighteen protein amino acids and two non-protein amino acids, dencichin and GABA, were found in samples with various steaming and drying periods. Amino acids can regulate pH values, while glutamic acid aids in synthesizing secondary ginsenosides [33]. The amount of protein and non-protein amino acids is regularly reduced as processing cycles increase.

### Principal component analysis

**Fig. 5:**
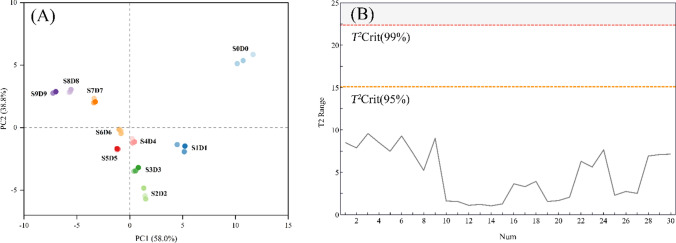
Score of principal component analysis of each ingredient of black ginseng "nine steaming and nine sun-drying" samples in ten batches (**A**), Hotelling *T*.^2^ test (**B**)

The processed datasheet containing the HPLC results of 30 ginseng samples was imported into SIMCA 14.1 software for PCA analysis to examine the overall distribution of the sample data. According to Fig. 5A, which depicts the principal component analysis of the significant chemical ingredients from S0D0 to S9D9, PC1 and PC2 contributed 58.0% and 38.8%, respectively, for a total of 96.8%. It was found that the two principal components were responsible for most of the chemical information in the black ginseng samples. Initial distinctions can be made between groups of samples: S0D0 had the giant projection on PC1 and PC2 and was located in the first quadrant, while S7D7, S8D8, and S9D9 were in the second quadrant; S1D1 and S2D2 were in the fourth; and S4D4, S5D5, and S6D6 were relatively close to the coordinate axes, which indicated that there was a considerable difference between chemical ingredients of samples in various steaming and sun-drying cycle periods. The absence of outliers in the sample data was further verified by the Hotelling *T*^*2*^ test (Fig. 5B). The content of each secondary ginsenoside and primary ginsenoside of black ginseng altered dramatically during the processing. This was the main reason for the different positions of various black ginseng samples in the coordinate system.

### Orthogonal partial least squares discriminant analysis

**Fig. 6:**
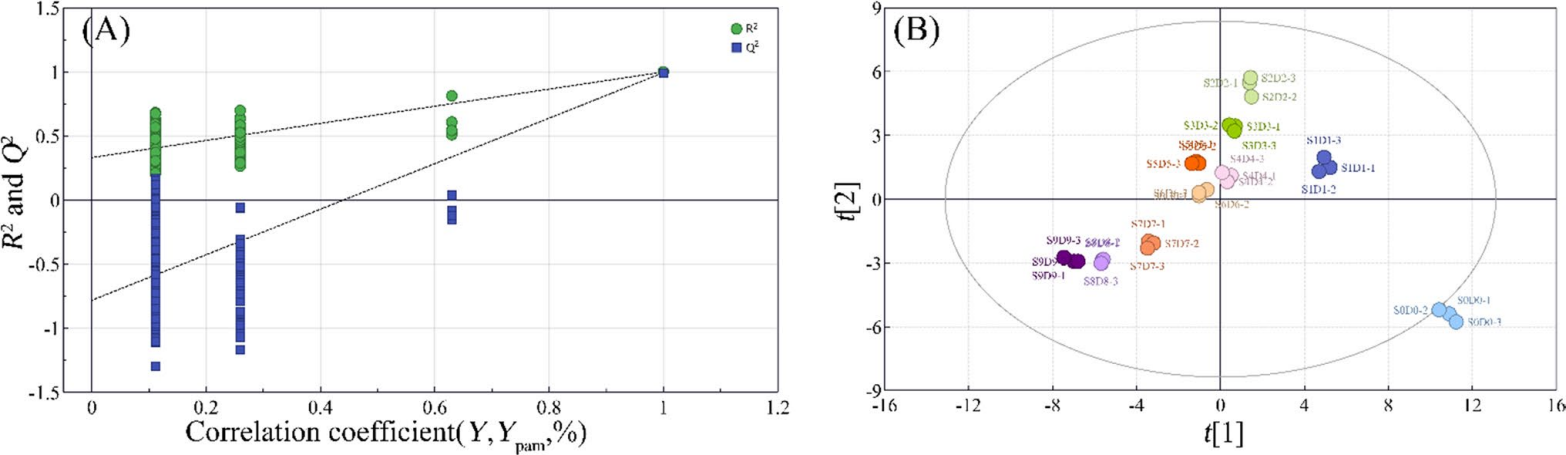
Model cross-validation results (**A**), OPLS-DA results of (**B**)

Ten batches of ginseng samples were analyzed using further processed HPLC data to build an OPLS-DA model. The differential ingredients of each sample could be effectively differentiated by utilizing the OPLS-DA analysis with SIMCA software. This analysis showed a fit index of 0.978 for the independent variable (R^2^x), 0.982 for the dependent variable (R^2^y), and 0.862 for the model prediction index (Q^2^), with R^2^ and Q^2^ exceeding 0.5 to indicate that the results of the model fit were acceptable. This shows that the model can describe most of the data and has good predictive ability. The validity of the model was further evaluated using the permutation test. An intersection between the Q^2^ regression line and the vertical axis was smaller than zero after 200 permutation tests, suggesting that the model was not overfitted and that it had been verified [34], and the results are shown in Fig. 6(A). The OPLS-DA score plot (Fig. 6(B)) showed that all the samples could be well differentiated according to the number of process times, suggesting some similarity in the chemical composition of black ginseng with the same cycle of steaming and sun-drying. In contrast, there was a difference between the ten samples with various steaming and sun-drying cycle periods. According to variable importance in the project (VIP) > 1 obtained by the OPLS-DA model, the ingredients with significant differences were screened out in Table 1. This suggests that they significantly affect the categorization of the different groups, while the other factors have a relatively small effect. Finally, the top 10 differential ingredients—fructose, glucose, ginsenoside 20 (S)-Rg3, 20 (R)-Rg3, 20 (S)-Rh2, dencichin, glutamic acid, ginsenoside Rg1, Re, and Rc—are filtered out.

**Table 1:** VIP value of OPLS-DA of each ingredient

Ingredient	VIP value	Ingredient	VIP value	Ingredient	VIP value
Fructose	1.38	citric acid	1.09	isoleucine	0.95
Glucose	1.34	L-malic acid	1.07	maltol	0.93
20 (S)-Rg3	1.34	tyrosine	1.05	GABA	0.92
20 (R)-Rg3	1.29	Rd	1.04	glycine	0.91
20 (S)-Rh2	1.24	acetic acid	1.03	5-HMF	0.91
Dencichin	1.23	Rb2	1.02	Rb3	0.90
Glutamic acid	1.16	20 (R)-Rh2	1.00	serine	0.89
Rg1	1.13	aspartic acid	0.98	Cysteine	0.89
Re	1.13	Rf	0.97	Malonic acid	0.89
Rc	1.13	proline	0.97	Maltose	0.88
Arginine	1.12	sucrose	0.97	Rg5	0.86
Methionine	1.11	D-malic acid	0.95	Rk1	0.86

### Network pharmacological analysis

**Fig. 7:**
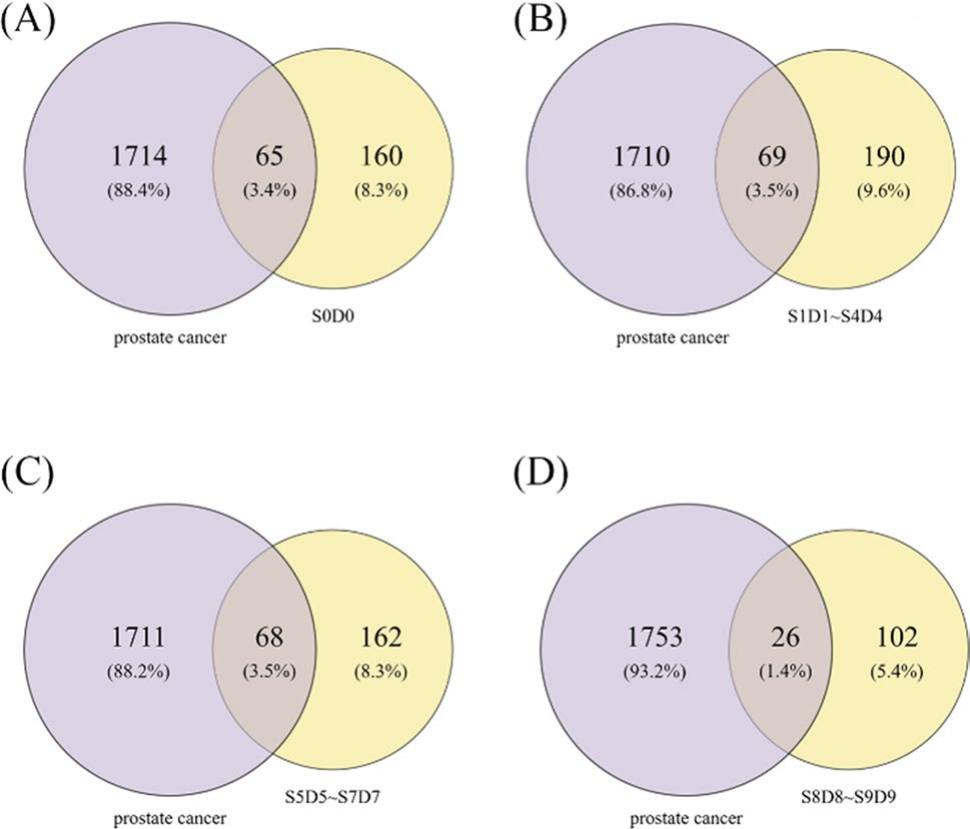
The Venn graph of potential targets in both black ginseng and prostate cancer: S0D0 (**A**), S1D1 ~ S4D4 (**B**), S5D5 ~ S7D7 (**C**), S8D8 ~ S9D9 (**D**)

**Fig. 8:**
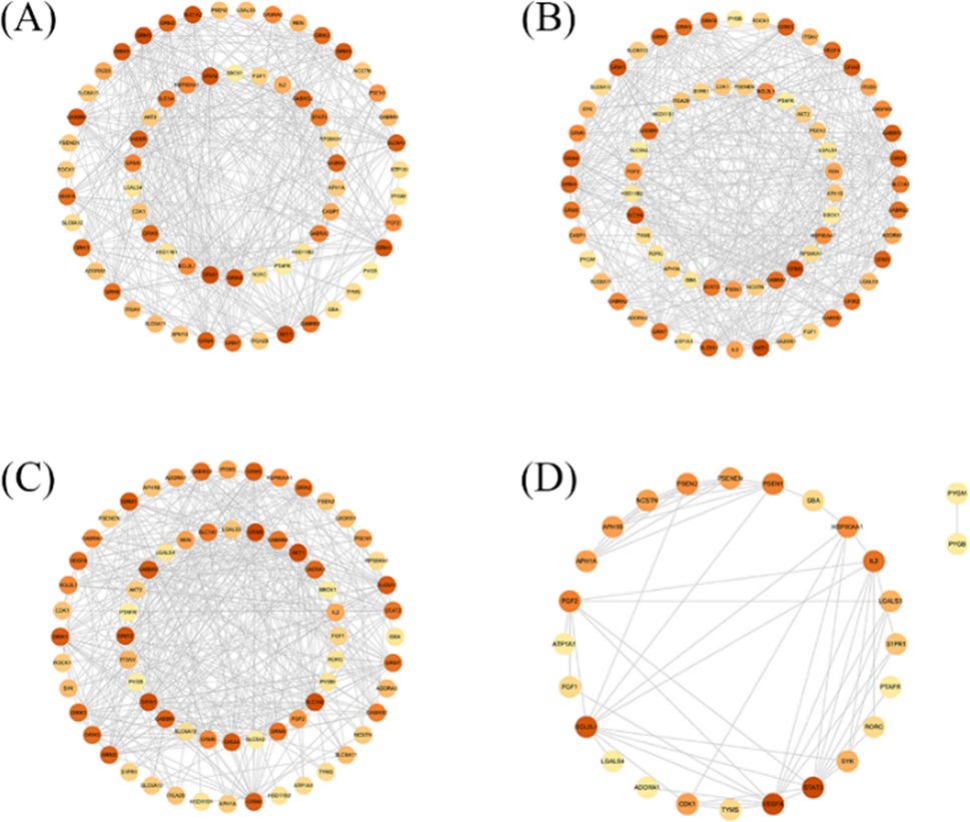
PPI network of the common targets of black ginseng and prostate cancer: S0D0 (**A**), S1D1 ~ S4D4 (**B**), S5D5 ~ S7D7 (**C**), S8D8 ~ S9D9 (**D**)

The SwissTargetPrediction and UniProt database was used to identify the targets of the ten differential ingredients mentioned above by qualifying the species as "Homosapiens". The genes with a more significant association with prostate cancer were screened by GeneCards and OMIM database, and 1779 high-scoring targets were obtained by combining and eliminating duplicate genes. Combined with the HPLC results, it can be seen that S1D1–S4D4, S5D5–S7D7, and S8D9–S9D9 contain the same differential ingredients, respectively. The targets of the differential ingredients contained in S0D0–S9D9 were combined, intersected with the disease targets, and plotted on the Venn diagram (Fig. 7) to obtain their effective targets. 65, 69, 68, and 26 intersected targets were obtained for S0D0, S1D1–S4D4, S5D5 –S7D7, and S8D9 –S9D9, respectively. The above-intersected targets were imported into the String database, and the species was limited to "human" to obtain the protein interactions network, which was further imported into Cytoscape for visualization and analysis. The Degree ranking of each target was obtained, and the results are shown in Fig. 8. Network nodes in the PPI network represent proteins, and edges represent protein–protein associations. The darker the node's color, the more associated it is with other proteins and has a higher degree value.

**Fig. 9:**
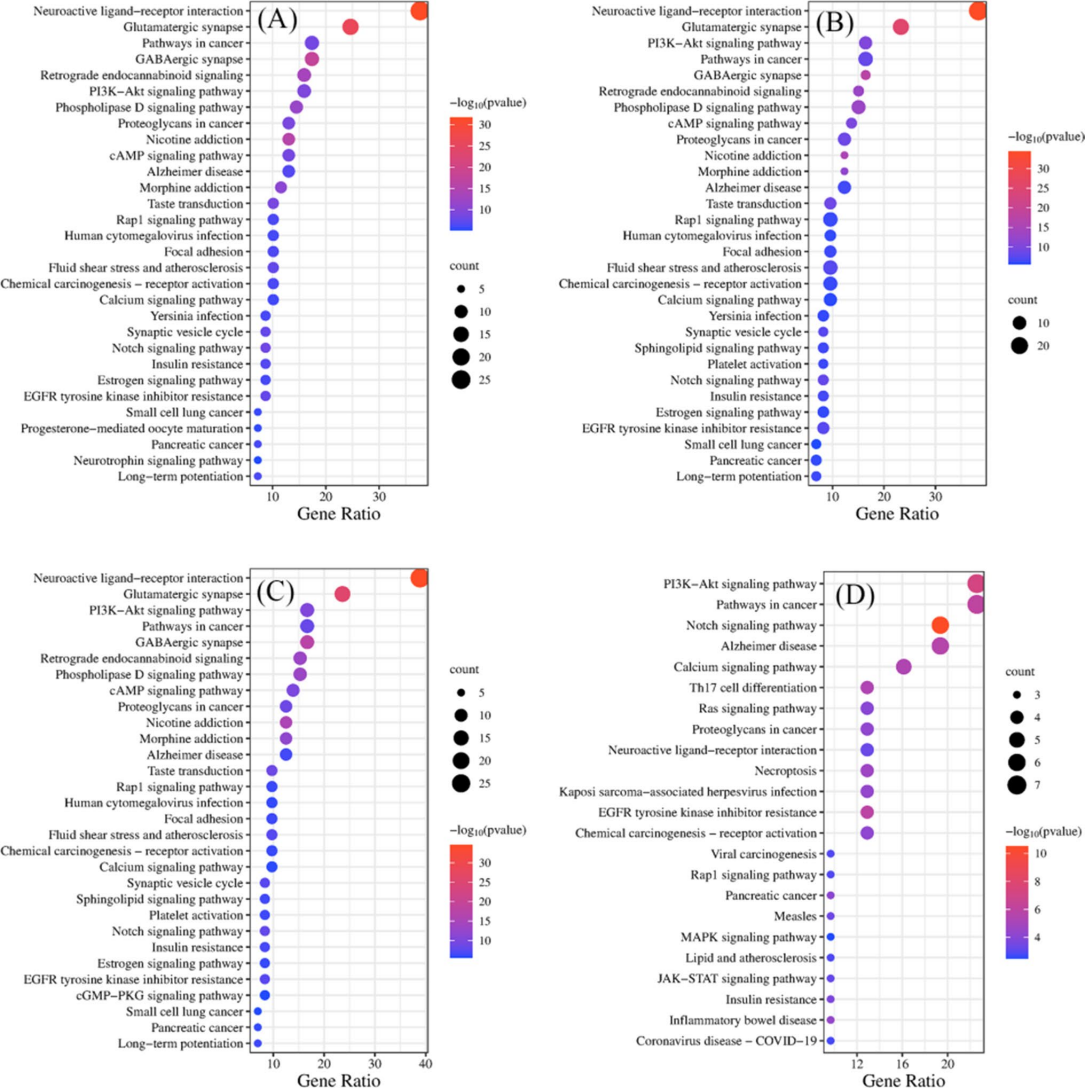
Results of KEGG pathway analysis of anti-prostate cancer in black ginseng samples: S0D0 (**A**), S1D1 ~ S4D4 (**B**), S5D5 ~ S7D7 (**C**), S8D8 ~ S9D9 (**D**)

The KEGG pathway annotation analysis of critical targets of S0D0 ~ S9D9 against prostate cancer showed that they were enriched with 115, 122, 122, 122, 122, 118, 118, 118, 31, 31 signaling pathways, respectively (Fig. 9). The enrichment results showed that the anti-prostate cancer effects of black ginseng samples during the "nine steaming and nine sun-drying" process mainly involved the neuroactive ligand-receptor interaction, glutamatergic synapse, GABAergic synapse, notch signaling pathway, PI3K-Akt signaling pathway, etc.

### Entropy weighting method assignment

**Fig. 10:**
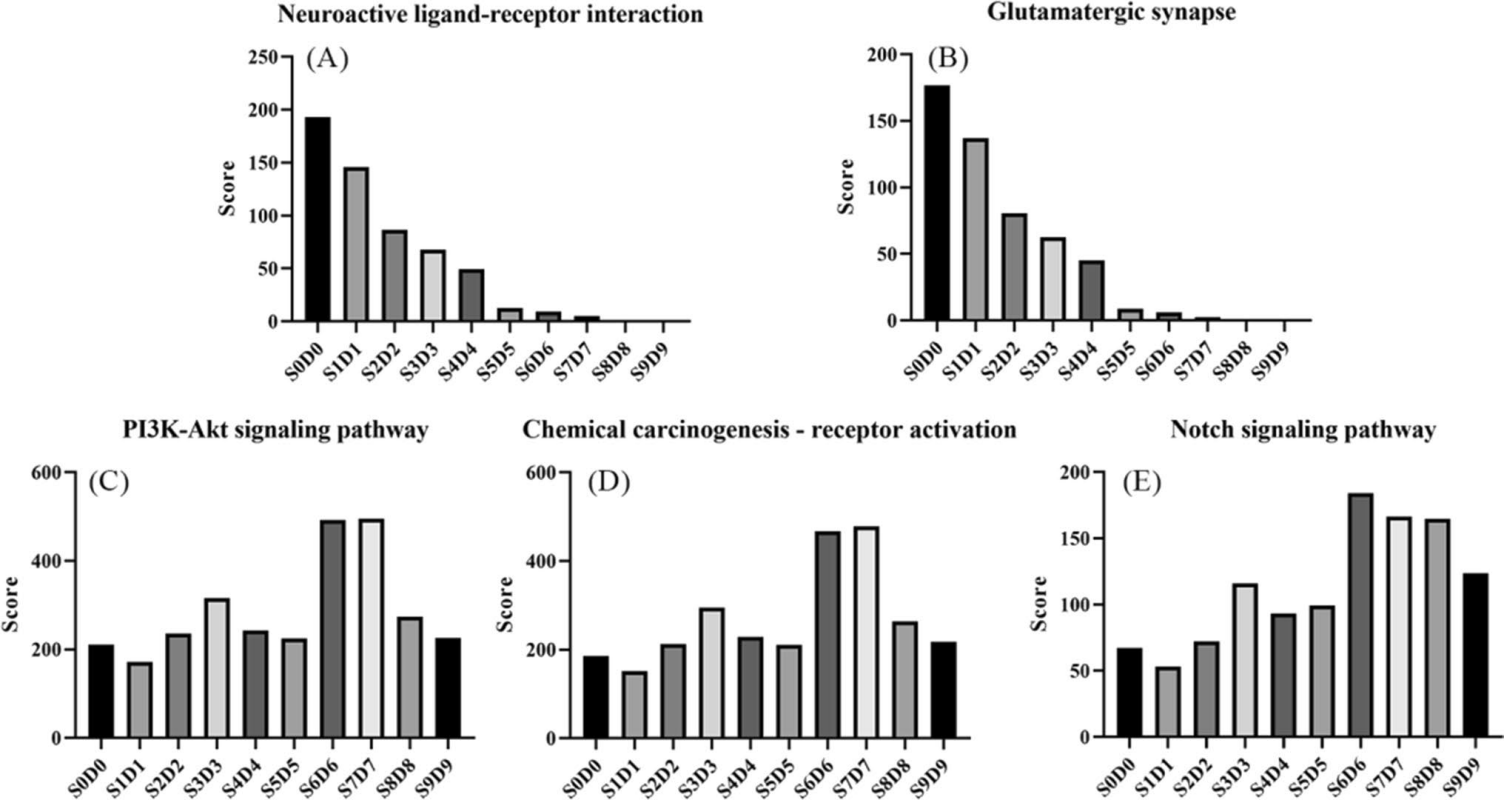
Effect of steaming and sun-drying times on black ginseng's anti-prostate pathway

Based on Eqs. 1 to 5, the final assigned value (*ρ*) of the ingredients in SnDn are obtained, and the results are shown in Table 2. According to Eqs. 6 to 8, the scores (*ω*) of the S0D0 ~ S9D9 anti-prostate cancer pathway were calculated. As is shown in Fig. 10, the scores of the neuroactive ligand-receptor interaction pathway (A), glutamatergic synapse pathway (B), PI3K-Akt signaling pathway (C), Chemical carcinogenesis-receptor activation pathway (D), and Notch signaling pathway (E) were changed regularly with the increase of the various steaming and sun-drying cycle periods. As the number of steaming and sun-drying cycles increased, the scores for pathways A and B steadily dropped, showing that the benefits of black ginseng on these two pathways faded as the number of processing cycles rose. The scores of pathways C, D, and E showed a tendency to increase and then decrease, and the scores of pathways C and D in S7D7 were the highest.

**Table 2:** Weights of the main differentiating ingredients (*ρ*)

Ingredient	S0D0	S1D1	S2D2	S3D3	S4D4	S5D5	S6D6	S7D7	S8D8	S9D9
Fructose	3.95	2.99	5.72	9.37	7.63	6.61	19.43	22.73	23.48	21.11
Glucose	0.97	0.62	1.12	2.20	1.97	2.07	4.34	3.70	4.68	3.62
20 (S)-Rg3	0	0.03	0.04	0.10	0.07	0.12	0.10	0.28	0.14	0.05
20 (R)-Rg3	0	0.06	0.07	0.11	0.10	0.11	0.11	0.30	0.14	0.07
20 (S)-Rh2	0	0.01	0.02	0.03	0.03	0.04	0.04	0.07	0.04	0.02
Dencichin	0.50	0.36	0.39	0.21	0.11	0.09	0.15	0.04	0	0
Glutamic acid	0.42	0.49	0.49	0.45	0.17	0.12	0.29	0.09	0	0
Rg1	1.72	1.47	0.87	0.67	0.46	0	0	0	0	0
Re	0.15	0.07	0.04	0.03	0.03	0.02	0.02	0.01	0	0
Rc	0.39	0.19	0.17	0.16	0.11	0.22	0.10	0.08	0	0

According to the HPLC data, dencichin, glutamate, Rg1, Re, and Rc content decreased in S0D0–S9D9 and reached zero in S8D8 and S9D9. Fructose and glucose concentrations rise gradually and steadily, but ginsenosides 20 (S)-Rg3, 20 (R)-Rg3, and 20 (S)-Rg2 concentrations fall after peaking in S7D7. Fructose and glucose had a particular promoting effect on prostate cancer, with 20 (S)-Rg3, 20 (R)-Rg3, and 20 (S)-Rh2 having a specific inhibitory effect that is greater than the promoting effect of fructose and glucose. Based on the results of HPLC analysis and vector space network pharmacology, we found a decrease in the content of dencichin, glutamate, Rg1, Re, and Rc, as well as an increase in the content of fructose, glucose, 20 (S)-Rg3, 20 (R)-Rg3, and 20 (S)-Rh2, weakened the regulation of black ginseng on the neuroactive ligand-receptor interaction pathway and glutamatergic synapse pathway, and enhanced its regulatory effects on the PI3K-Akt signaling pathway, chemical carcinogenesis-receptor activation pathway, and Notch signaling pathway. The variation in the content of different components in black ginseng during processing leads to its different regulatory effects on prostate cancer-related pathways, which is the main reason for the change in the anti-prostate cancer effect of black ginseng in various steaming and sun-drying cycle periods.

Carbohydrates are crucial components of cellular metabolism and may provide prostate cancer cells with more energy. The expression of glucose transporter 1 (Glut-1) in prostate cancer cells rises with the grade of the malignant tumor [35], while the fructose transporters Glut5 and Glut9 are markedly elevated in prostate cancer patients [36]. Ginsenoside is the main active ingredient in ginseng and black ginseng. Ginsenoside Rg3 inhibits the expression of AQP1 in highly metastatic PC-3 M prostate cancer cells to reduce their migration level [37]. Rg3 may also inhibit the cell cycle by increasing the ROS produced by prostate cancer cells [38]. Ginsenoside Rh2 can upregulate ROS, superoxide, and PPAR-δ; downregulation of p-STAT3 induces apoptosis in prostate cancer cells [39]. Many prostate cancer patients are associated with transmitting neural active ligand-receptor interaction signals [40]. The transmission of various glutamate receptors, such as mGluRs and iGluRs, is involved in cell proliferation and migration in cancer [41]. The activation of PI3K/Akt in the PI3K/Akt pathway enhances the levels of Bcl-2 and XIAP, thereby improving the survival rate of prostate cancer cells [42]. PI3K/Akt may also work together with other pathways to worsen the condition of prostate cancer[43]. The activation of Erk/MAPK signals in the chemo-carcinogenic receptor activation pathway is the mechanism of cadmium-induced prostate cancer [44]. Notch signaling is highly expressed in prostate cancer, and its expression increases with the cancer grade [45, 46]. These accounts concur with the findings of our experiment. Therefore, vector space network pharmacology methods can accurately and effectively analyze the anti-prostate cancer mechanism of black ginseng.

## Conclusion

Traditional Chinese Medicine (TCM) has the characteristics of diverse chemical components and complex effects, making it challenging to describe its therapeutic mechanism. Most current research ignores the multi-component and multi-target aspects of TCM in favor of explaining its effectiveness from the standpoint of its monomers. This study was based on the HPLC content determination results of black ginseng samples with different processing times and combined multivariate statistical analysis, network pharmacology, and the entropy weight method to establish a vector space network pharmacology analysis method.

In this study, the contents of six organic acids, maltol, 5-HMF, 17 ginsenosides, four oligosaccharides, and 20 amino acids were determined based on the quantitative HPLC analysis of the aqueous extracts of ten batches of black ginseng samples from the "nine-steaming and nine sun-drying" process. It was found that the content of primary ginsenosides was gradually converted to secondary ginsenosides, oligosaccharides were gradually hydrolyzed to monosaccharides, the content of 5-HMF and maltol tended to increase, and the content of protein and non-protein amino acids were gradually decreased during the steaming and sun-drying process of black ginseng. Ten differential black ginseng components were chosen through content determination and multivariate statistical analysis, and a vector space network study on anti-prostate cancer was performed. It was found that with the increase in the number of cycles of steaming and sun-drying, the contents of fructose, glucose, 20 (S)-Rg3, 20 (R)-Rg3, and 20 (S)-Rh2 were elevated, and the contents of Rg1, Re, dencichin, glutamate, and Rc were decreased. This change led to the weakening of the effects of black ginseng on the neuroactive ligand-receptor interaction pathway and glutamatergic synaptic pathway and the enhancement of the effects on the PI3K-Akt signaling pathway, the chemo-carcinogenic receptor activation pathway, and the Notch signaling pathway. That was the primary mechanism of black ginseng’s anti-prostatic cancer effect during the "nine-steaming and nine sun-drying" process.

In summary, this study indicated that the vector space network pharmacology methods based on HPLC analysis could effectively elucidate the potential mechanism of anti-prostate cancer action of black ginseng during the "nine steaming and nine drying" process. Moreover, vector space network pharmacology introduces the concept of "quantity", which is no longer limited to the analysis of a single sample of TCM but applies to the analysis of similar TCM with the same chemical composition but varied contents. It can provide a feasible reference model for the screening of efficacy ingredients and the traceability of the mechanism of action in the course of herbal processing.

## Data Availability

The datasets generated during and analyzed during the current study are available from the corresponding author upon reasonable request.
